# Efficacy of a Postbiotic Formulation Combined With Microneedling for Mild‐to‐Moderate Acne: A Self‐Control Study

**DOI:** 10.1111/jocd.16703

**Published:** 2024-12-02

**Authors:** Zhanhong Li, Peihui Li, Yu Xu, Changqing Yan, Xiufen Ma, Huiying Wang, Hong Cheng, Jing Zeng, Ting Li, Xinxian Li, Jia Zhou, Jie Zhang, Jianfeng Zhou, Rongya Yang, Yan Wu, Li Li, Wei Lai, Jiangyun Zhao, Zhe Liu, Qiong Meng

**Affiliations:** ^1^ Guangzhou MLT Medical Cosmetic Clinic Guangzhou China; ^2^ Changsha 7HE VLINES Medical Cosmetic Hospital Changsha China; ^3^ Wuhuazhenqi Medical Cosmetic Clinic Kunming China; ^4^ Non‐Surgical Center, Changsha MYLIKE Medical Cosmetic Hospital Changsha China; ^5^ Department of Cosmetic Dermatology Xiamen MYLIKE Medical Cosmetic Hospital Xiamen China; ^6^ Department of Traditional Chinese Medicine Cosmetic Dermatology Shenzhen Yestar Medical Cosmetic Hospital Shenzhen China; ^7^ Beijing J Yan LaserKing Medical Cosmetic Clinic Beijing China; ^8^ Department of Cosmetic Dermatology Fuzhou Maen Medical Cosmetic Clinic Fuzhou China; ^9^ Medical Cosmetic Center Xinjiang Uiger Municipal People's Hospital Urumqi China; ^10^ Shenzhen Coastal Starlight Medical Cosmetic Clinic Shenzhen China; ^11^ Department of Dermatology the Seventh Medical Center of Chinese PLA General Hospital Beijing China; ^12^ Department of Dermatology Peking University First Hospital Beijing China; ^13^ Department of Dermatology West China Hospital of Sichuan University Chengdu China; ^14^ Department of Dermatology The Third Affiliated Hospital of Sun Yat‐Sen University Guangzhou China; ^15^ Beijing Heyan Yijie Medical Beauty Clinic Beijing China; ^16^ Shenyang Heping Heyan Xiandao Medical Beauty Clinic Shenyang China

**Keywords:** acne vulgaris, microneedling, postbiotics, skin barrier, skin microbiota

## Abstract

**Background:**

Acne vulgaris significantly affects young individuals globally, with its onset associated with an increased prevalence of C acnes, a naturally occurring skin bacterium. In light of the rising concerns regarding antibiotic resistance and the potential for adverse effects, pharmacological interventions may not consistently represent the most suitable option. Nonpharmacological approaches, such as microneedling, offer promising alternative treatment modalities. Furthermore, the integration of postbiotics in skincare formulations for acne management has gained traction recently. Nonetheless, there remains a lack of sufficient evidence to establish the efficacy and safety of postbiotics when combined with microneedling.

**Objective:**

To assess the clinical effectiveness of a postbiotic formulation combined with microneedling in mild‐to‐moderate facial acne vulgaris.

**Methods:**

Twenty Chinese patients were enrolled, all received one treatment and were monitored 4 weeks posttreatment. The evaluation assessed symptom improvement, treatment safety, and patient satisfaction.

**Results:**

The global acne grading system (GAGS) score decreased more than half based on the baseline. Clinical photographs following treatment revealed improved the skin lesions and brightened skin tone. Statistics from VISIA showed excellent improvement in speckle, pore, red region, and porphyrin. No significant adverse reactions have been reported during the treatment period. Additionally, more than 85% were very satisfied or satisfied with the outcome.

**Conclusion:**

Our results showed that the postbiotic formulation combined with microneedling may benefit the restoration of the skin barrier and the equilibrium of skin microbiota. This approach may help mitigate inflammation and address skin lesions, presenting a promising therapeutic avenue for the prevention and management of acne vulgaris.

## 1 Introdution

Acne vulgaris is a chronic inflammatory disease of the pilosebaceous unit that impacts youth worldwide, leading to considerable psychological and social difficulties in their daily lives [1, 2]. The pathogenesis of acne is complex, sebum production, hyperkeratinization of the pilosebaceous infundibulum, dysbiosis of the skin microbiota, and abnormal inflammation are recognized as the four major developmental processes of acne [3, 4]. The standard therapeutic approach for managing acne typically encompasses the application of topical ointments, frequently combined with oral medications, such as antibiotics (clindamycin and erythromycin), topical retinoids, and oral isotretinoin [5, 6]. However, medications are not always effective, and even have potential side effects, including drug resistance and skin sensitization [7, 8].

A vast number of microorganisms, estimated in the trillions, inhabit the surface of human skin that the dynamic equilibrium between long‐term resident bacteria and short‐term resident bacteria helps maintain the skin barrier and immune function [9]. Recent research has indicated a positive correlation between the extent of modifications in skin microbiota and the severity of acne, suggesting the microbiota's involvement in the progression of acne [10, 11, 12]. It is reported that hyperproliferation of C acnes and S aureus, as well as their secretion of CAMP and proteases, promote the development of acne by disrupting the skin barrier and inducing inflammation [13]. Therefore, regulating skin microbiota and restoring skin microecological homeostasis has become a new concept in acne treatment.

Recently, postbiotic was defined by the International Scientific Association of Probiotics and Prebiotics as “preparations of inanimate microorganisms and/or their components that confer a health benefit on the host,” displaying properties of anti‐inflammatory, immunomodulatory, antioxidant, and superior safety, which show great potential in acne treatment [14, 15, 16]. Lactobacillus fermentation lysate is the main functional ingredient of EVE‐CHARM antiacne lotion used in this study, which has been proven to have cosmetic effects such as restoring the skin barrier, anti‐inflammatory, and inhibiting melanin production [17, 18]. Nevertheless, the successful administration of topical formulations to the deeper layers of the skin is essential for optimizing their therapeutic effectiveness. Microneedles possess the capability to penetrate the skin and create microchannels, thereby offering a highly effective drug delivery method that has the potential to significantly improve the permeation and absorption of topical formulations [19]. Herein, we aimed to examine the efficacy and safety of EVE‐CHARM antiacne lotion combined with microneedling in the treatment of mild‐to‐moderate acne.

## 2 Subjects, Materials and Methods

### 2.1 Subjects

The inclusion criteria for this study were established as follows: participants aged between 18 and 30 years, diagnosis of Grade I–III facial acne vulgaris in accordance with Chinese acne management guidelines, in good health based on physical examinations and laboratory test results, exhibiting facial symmetry in the treatment area, and written consent to participate [20]. The study excluded individuals with underlying medical conditions, those with active acne or facial infections, pregnant or lactating females, patients with visible wounds and scar formation tendencies, other inflammatory and infectious skin diseases. A total of 20 healthy individuals successfully fulfilled the eligibility criteria and were enrolled in the study from June 2023 to March 2024 in Guangzhou MLT Medical Cosmetic Clinic. Ethical approval was granted by the ethics committee of Guangzhou MLT Medical Cosmetic Clinic (No. 2023002). All participants were duly informed of the study procedures and provided their signatures on the requisite written consent forms.

### 2.2 Materials

#### 2.2.1 Rolling Microneedle

The skin rolling microneedle (Guangdong Food and Drug Administration Approval No. 20222200879) was provided from Guangzhou LTHINK Biotechnology Co. Ltd. (Guangdong, China). Specification: 0.22 mm (needle diameter) × 0.5/1.0 mm (needle length), 192 needles, sterile single‐use.

#### 2.2.2 Postbiotic Formulation

The postbiotic formulation (EVE‐CHARM antiacne lotion, Guangdong Cosmetics Approval No.20161582) was provided from Jishengyuan Biotechnology Co. Ltd. (Guangzhou, China), which is composed of two parts: the lyophilized powder and the accompanying solution, as shown in Table 1. Before application, the lyophilized powder should be thoroughly mixed with the accompanying solution (pH 6.8 for the mixture), and then the mixture should be applied to the face immediately.

**TABLE 1:** List of ingredients in postbiotic formulation.

	The lyophilized powder	The accompanying solution
Main ingredients	Water, mannitol, Lactobacillus fermentation lysate, trehalose, copper tripeptide‐1	Water, allantoin, propylene glycol, panthenol, sodium hyaluronate
Trace ingredients	Acetyl Hexapeptide‐7, 1,2‐pentanediol, oligopeptide‐1, hexapeptide‐9, p‐hydroxyacetophenone, hexanediol, glycine, alanine, proline, glycerol, hydrogenated lecithin, ceramide NP	Methylparaben

### 2.3 Methods

#### 2.3.1 Clinical Data

In this study, we collected clinical data from 3 male and 17 female patients in the age group of 22–30 years with facial acne vulgaris who received treatment by EVE‐CHARM anti‐acne lotion combined with microneedling at Guangzhou MLT Medical Cosmetic Clinic from May 2023 to March 2024. Included were patients (1) who fulfilled the diagnostic criteria for Grade I–III facial acne vulgaris, (2) who had received one combined treatment with microneedling and EVE‐CHARM antiacne lotion, and (3) with comprehensive clinical and follow‐up data. Individuals who underwent additional facial cosmetic surgeries and treatments, including but not limited to laser therapy, radiofrequency procedures, or injectable treatments, within a 6‐month timeframe preceding, concurrent with, or following the designated treatment period, were excluded from participation. Furthermore, individuals utilizing medications that may potentially impact the outcomes of the study, such as retinoic acid or hydroquinone, were also excluded from the participant cohort.

#### 2.3.2 Preoperative Preparations

Prior to the commencement of the treatment, participants were provided with a comprehensive overview of the entire treatment protocol as well as postoperative care guidelines. They were required to sign informed consent forms pertaining to both the procedure and the associated research. The treatment area was thoroughly sanitized prior to the procedure, and photographic documentation was captured for record‐keeping purposes, with data meticulously organized in accordance with established standards. Subsequently, prior to the treatment, participants were administered facial anesthesia in alignment with aseptic principles, after which microneedle therapy was conducted under proper anesthesia.

#### 2.3.3 Intraoperative Operations

Different needle lengths target different levels of skin. The 0.5 mm length microneedle can reach the epidermal layer and basement membrane zone of the skin, while the 1.0 mm length microneedle targets the dermis layer. The appropriate length of microneedle should be selected based on facial skin zoning and the severity of acne. The needle was inserted perpendicularly into the skin, unordered scrolling operation several times. Diffuse erythema with exudation of tissue fluid as the endpoint of treatment. Immediately after microneedling, apply the EVE‐CHARM antiacne lotion to the acne lesion areas (if there are obvious blood stains, 0.9% saline can be used to rinse before operation).

#### 2.3.4 Postoperative Care

Subsequent to the completion of the treatment, medical cold patches were administered for the purpose of providing cold compresses. Patients received guidance to refrain from washing their faces or engaging in any stimulating activities for a duration of 3 days following the treatment. Upon the conclusion of this specified timeframe, patients were permitted to resume their standard skincare regimen, with the recommendation to utilize gentle and safe moisturizing products. To ensure adequate protection of their skin against sun exposure, patients were strongly encouraged to incorporate the use of sunscreen or appropriate protective equipment, such as hats and masks, into their daily activities.

### 2.4 Assessment

Standardized facial photographs were obtained during each appointment utilizing a VISIA imager system and a SLR camera. The efficacy of combined treatment with microneedling and EVE‐CHARM antiacne lotion improvement was assessed through the global acne grading system (GAGS) by experienced dermatologists [21]. The GAGS score was based on semi‐quantitative, assessing comprehensively from various aspects, including the number and coverage of pimple, papule, pustule. The curative effect was assessed through the Symptom Score Reduction Index (SSRI), which is calculated using the following formula: SSRI = (total score before treatment—total score after treatment)/(total score before treatment) × 100%. Values of 0%, 1%–25%, 26%–50%, 51%–75%, and 76%–100% indicated no, mild, moderate, significant, and excellent. Adverse reactions experienced by participants were meticulously recorded, and a follow‐up was performed 4 weeks posttreatment. Patients' satisfaction was given at a 4‐week follow‐up with a five‐category scale.

### 2.5 Statistical Analysis

Data statistical analysis and bar plot generation were conducted using SPSS version 22.0 (IBM Corporation, USA) and GraphPad Prism version 8.0 (GraphPad Software Inc., USA). To evaluate the differences in outcomes prior to and following treatment, Student's test (*t*‐test) was applied. The *p* value < 0.05 (*p* < 0.05) was deemed statistically significant. Results were presented as mean ± SEM.

## 3 Results

### 3.1 Patients' Characteristics

A retrospective analysis was conducted on the data of 20 patients, comprising 17 females and 3 males. The age of the patients varied from 22 to 30 years old (25.28 ± 2.46). Based on the Chinese guidelines for the management of acne vulgaris, 9 patients were diagnosed as grade II (45%), and 11 patients were diagnosed as grade III (55%).

### 3.2 Efficacy

**FIGURE 1:**
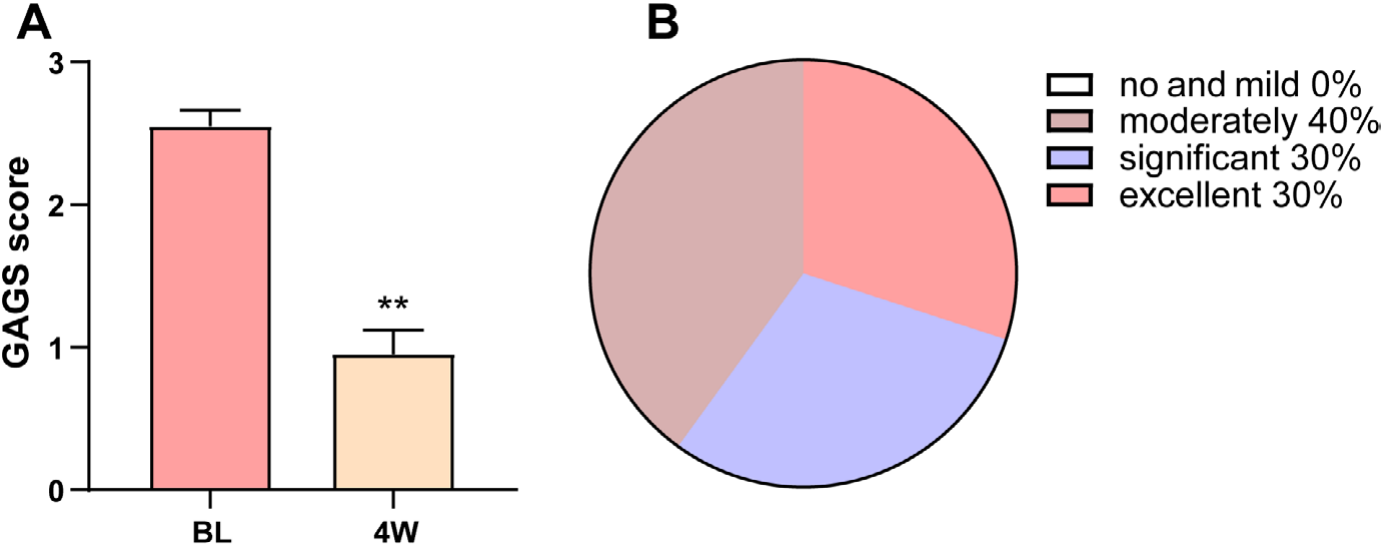
Treatment of EVE‐CHARM antiacne lotion with microneedling improved acne. (A) The GAGS score from baseline and 4 weeks after combined treatment with EVE‐CHARM antiacne lotion and microneedling. (B) Analysis results of improvement effects. **p <  0.01 compared with BL. 4 W, 4 weeks; BL, baseline.

**FIGURE 2:**
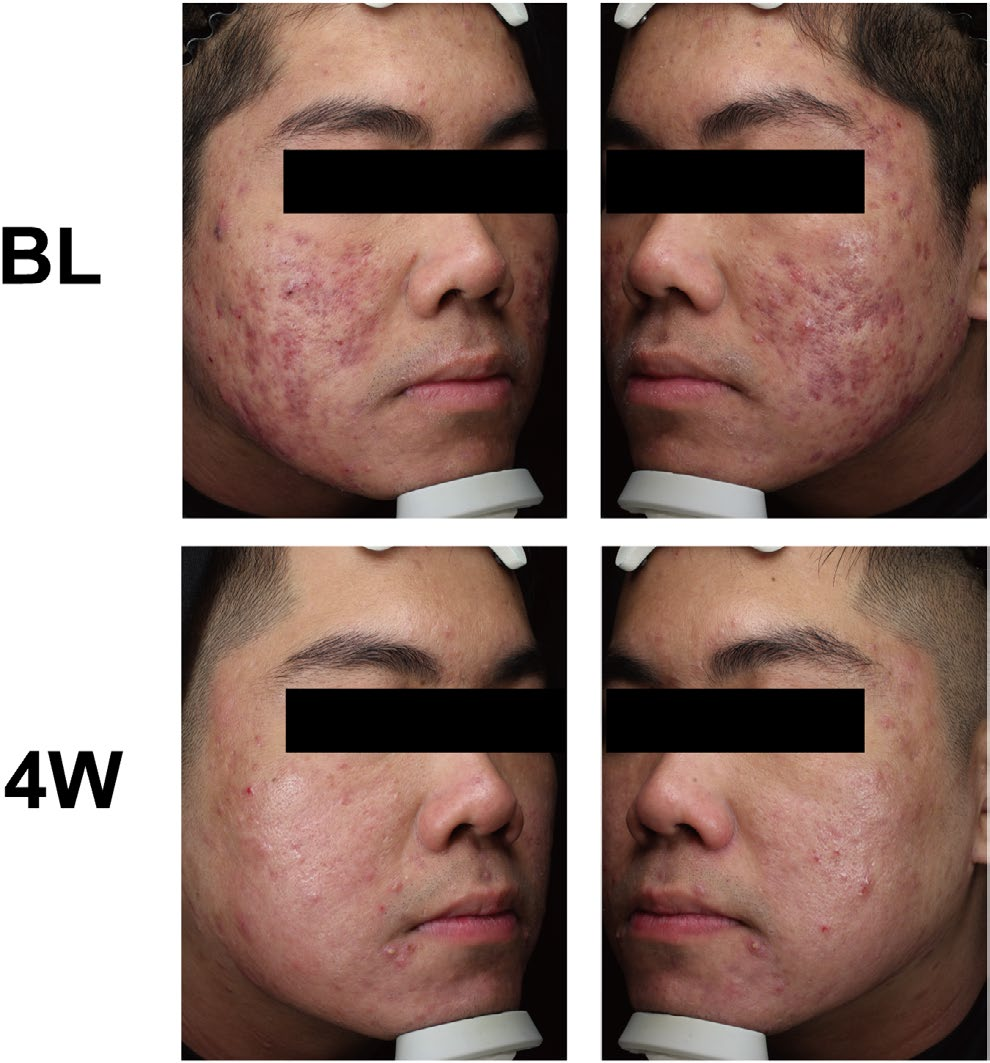
Representative photos before and after treatment. This 23‐year‐old male patient has experienced acne vulgaris since his adolescent years. After one combined treatment with microneedling and EVE‐CHARM antiacne lotion, lesions were improved on Week 4. BL, baseline; 4 W, week 4.

Professional doctors use GAGS to evaluate the improvement of acne in patients, which is the most widely applicable acne assessment method. The GAGS score assessment is as follows: a score of 0 indicates the absence of acne lesions, although residual pigmentation and erythema may be present; a score of 1 signifies minimal acne, characterized by a few scattered open or closed comedones and very few papules; a score of 2 reflects mild acne, with < 50% of the facial area affected by several comedones, papules, and pustules; a score of 3 denotes moderate acne, where more than 50% of the facial area is affected, exhibiting numerous papules, pustules, comedones, and a maximum of one nodule. Compared with the baseline, the GAGS score was significantly decreased after combined treatment with microneedling and EVE‐CHARM antiacne lotion (Figure 1A). The effectiveness of the treatment was evaluated utilizing the SSRI formula, referencing GAGS scores obtained prior to and following the intervention period. Of the total 20 patients, 6 patients (30%) were excellent improved, 6 patients (30%) were significantly improved, and 8 patients (40%) were moderately improved (Figure 1B). Representative case photos are shown in Figure 2, a 23‐year‐old male patient, previously diagnosed with moderate acne, exhibited substantial improvement after 4 weeks of combined treatment with microneedling and EVE‐CHARM anti‐acne lotion that obviously refined pore appearance, reduced sebaceous gland activity, enhanced the condition of acne lesions including pimples and papules, and contributed to a more luminous skin tone.

### 3.3 Objective Assessment

**FIGURE 3:**
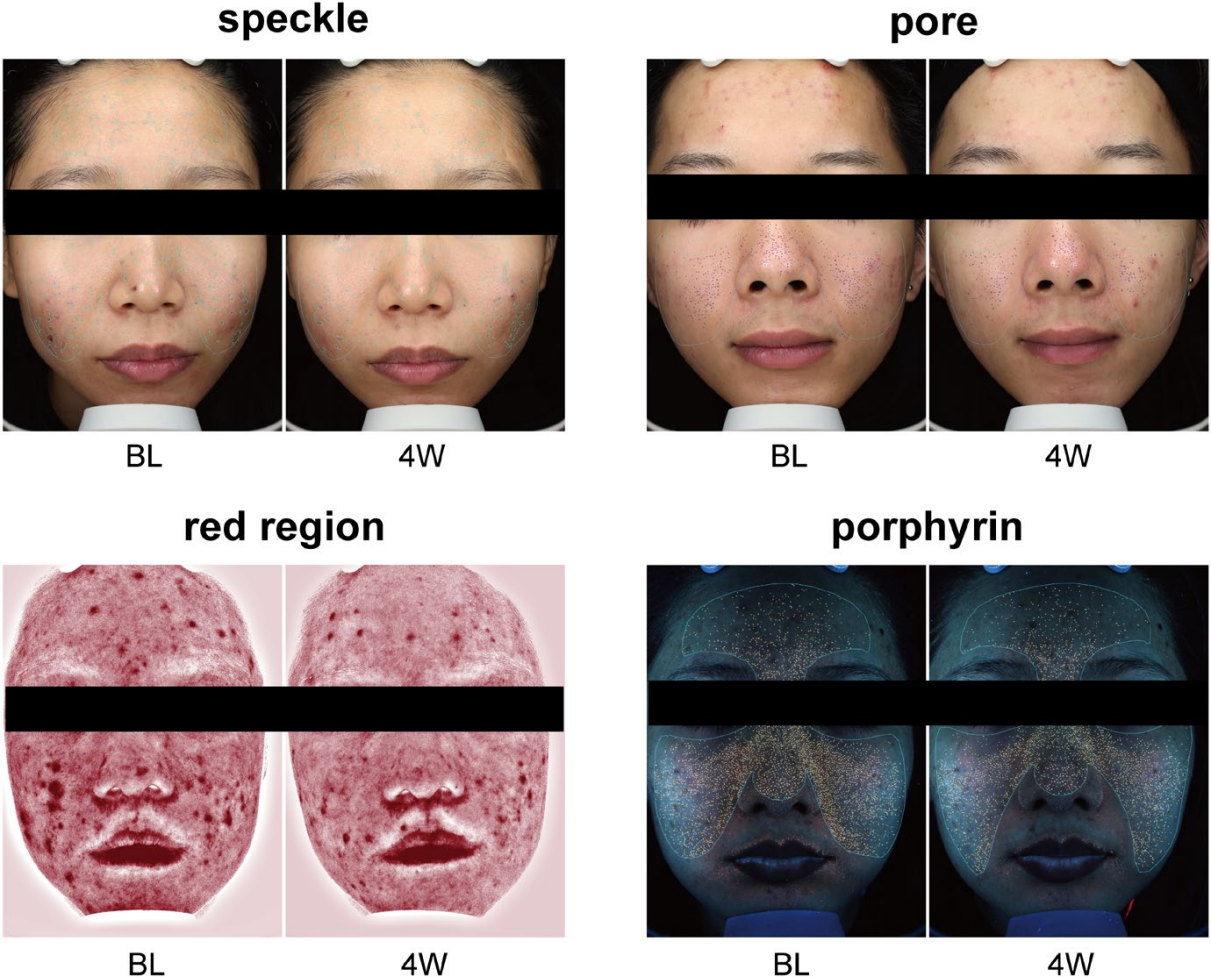
Representative photographs of before and after treatment comparisons recorded in the VISIA imager from speckle, pore, red region, and porphyrin items. BL, baseline; 4 W, week 4.

**FIGURE 4:**
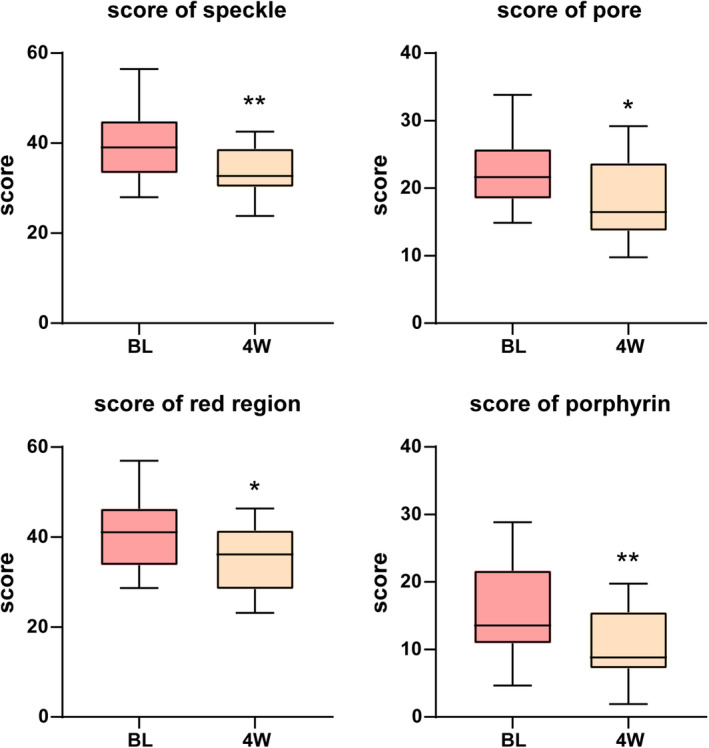
Mean score of speckle, pore, red region, and porphyrin recorded in VISIA imager. *p <  0.05, **p <  0.01 compared with BL. BL, baseline; 4 W, week 4.

Facial photographs of patients were captured utilizing the VISIA camera both prior to and following treatment, and a range of indicators were meticulously documented. The condition of visible skin lesions, inflammation, sebaceous gland secretion, and skin microbiota can be evaluated by speckle score, pore score, red region score, and porphyrin score. As shown in Figures 3 and 4, the above indicators showed significant improvement after combined treatment with microneedling and EVE‐CHARM antiacne lotion. Those evidence indicated that this topical formulation probably restores the balance of skin flora, strengthens the skin barrier, and alleviates inflammation to improve acne.

### 3.4 Patients' Subjective Assessment of Efficacy

**FIGURE 5:**
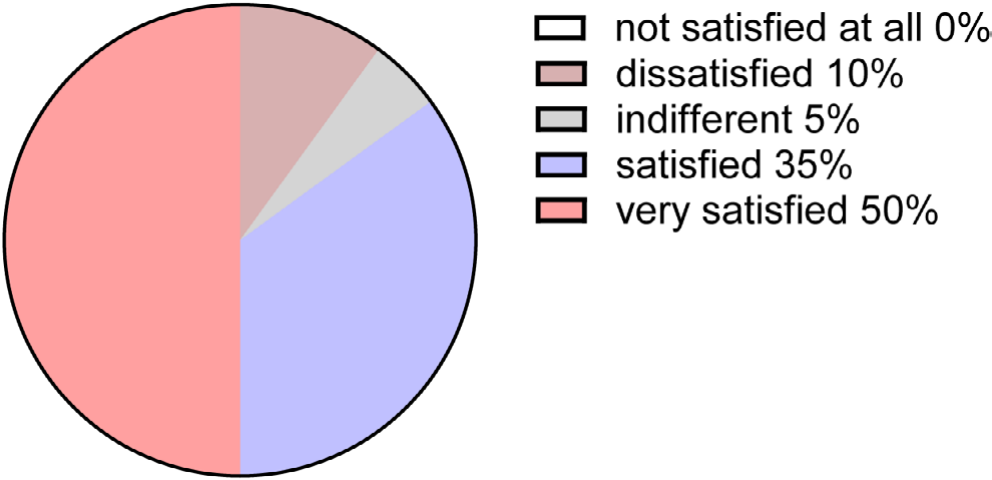
Proportion of patient satisfaction levels at the conclusion of Week 4.

Patients were invited to evaluate their level of satisfaction at a 4‐week follow‐up with a 5‐point scale: (1) not satisfied at all = 1; (2) dissatisfied = 2, (3) indifferent = 3, (4) satisfied = 4, (5) very satisfied = 5. The average score was 4.26 ± 0.99. Among the total cohort of 20 patients, 10 patients (50%) expressed a high level of satisfaction, categorizing their experience as very satisfied with the improvements observed. Additionally, seven patients (35%) reported being satisfied, while one patient (5%) indicated an indifferent stance, and two patients (10%) conveyed dissatisfaction regarding the outcomes (Figure 5). Consequently, it can be concluded that a significant majority of patients expressed a favorable perception of the improvement of EVE‐CHARM antiacne lotion combined with microneedling.

### 3.5 Adverse Reactions

Following the application of the roller microneedle, all participants exhibited a transient erythema of the skin surface. Additionally, a number of participants described experiencing a mild sensation akin to a needle prick during the procedure; however, this discomfort was deemed manageable. Postoperative follow‐up assessments indicated that the majority of participants achieved full recovery within 1‐week postprocedure, with no noteworthy adverse reactions reported.

## 4 Discussion

Acne is a prevalent chronic skin disease characterized by a significant incidence rate. If not effectively treated, it is easy to leave stubborn skin lesions, pigmentation, or scars, which exerts an adverse effect on the overall well‐being of the patient, encompassing both physical and mental health dimensions [22]. In light of the advancements in sequencing technologies, we have achieved considerable progress in comprehending the composition of the skin microbiota and the implications of ecological dysregulation on skin health [23]. More and more studies indicated that skin microecology significantly contributes to the pathogenesis of acne [24, 25]. The interplay between the skin microbiota, the host organism, and the external environment collectively forms the foundation of skin microecology [26]. The skin microbiome consists of two primary categories of microorganisms. The resident microbiome represents a stable and core population that possesses the ability to self‐replenish after experiencing any disturbances The transient microbiome, on the other hand, has a microbiome that does not permanently reside on the skin but appears for a few hours or days depending on the environment. In a state of optimum dermal health, both categories of microbiomes exist harmoniously within human skin, collaboratively preserving a dynamic equilibrium. This interplay facilitates the skin's barrier functions through a multifaceted approach, engaging in physical, chemical, microbial interactions, as well as both innate and adaptive immune responses [27]. However, the ongoing influence of various external and internal factors has a significant impact on the equilibrium of this system. Hyperkeratosis of follicular sebaceous gland duct cells and increased sebum volume combine to provide a relatively anaerobic and lipid‐rich environment for bacteria [28]. C acnes and S aureus proliferate extensively and become dominant bacteria, stimulating the release of corticotropin releasing hormone (CRH) to induce sebum synthesis and increase sebum secretion, which blocks the hair follicle sebaceous gland ducts [13]. Besides, C acnes can activate autoimmunity that induces differentiation of CD4^+^ T cells into Th17 cells and promotes secretion of inflammatory factors [29]. Thus, microbiota dysbiosis promotes the development of acne by weakening the skin barrier and activating the immune system.

In recent years, microecological therapy based on improving the microbiota balance of skin, restoring the skin barrier, and regulating immunity has become a new concept in acne treatment [30, 31]. Numerous clinical trials are currently being conducted to investigate the efficacy and potential adverse effects of microbial preparations derived from probiotics or their metabolites for the treatment of acne [32, 33]. Topical application of a probiotic *Lactobacillus plantarum* GMNL6 in acne individuals has the potential to significantly enhance collagen production and promote the expression of the gene associated with serine palmitoyltransferase small subunit A. Furthermore, this application may lead to a reduction in melanin synthesis, the prevalence of *Staphylococcus aureus* biofilm, and the proliferation of C acnes. Ultimately, these effects may contribute to improved skin hydration, complexion, pigmentation irregularities, wrinkle appearance, and porphyrin levels [17]. Compared with probiotics, postbiotics as formulations of inanimate microorganism, are defined as the organisms and metabolites of probiotics, which has the following advantages: (1) higher safety and extended product shelf life; (2) smaller active molecules, more easily absorbed by the skin; (3) act directly on the skin without being repelled by competition from harmful bacteria; (4) controlled preparation and more stable performance [34, 35]. Because of its multiple benefits to the skin without serious side effects, postbiotics become a promising ingredient for cosmetology and skincare.

In this study, it is the first time that the therapeutic efficacy of EVE‐CHARM anti‐acne lotion, a topical postbiotic formulation, combined with microneedling in mild to moderate acne vulgaris has been explored (Table 2). Utilizing microneedles to penetrate the skin surface in a minimally invasive manner to create multiple reversible micron‐sized channels to enhance drug penetration [36]. Moreover, microdamage caused by microneedling can unblock blocked follicle‐sebaceous gland duct channels and initiate skin repair mechanisms to promote metabolism and collagen regeneration [37]. We employed microneedling as a method to administer the postbiotic, resulting in favorable outcomes. The syndromes of skin lesions, pores, and inflammation were reduced to varying degrees after one treatment. After treatment, all patients demonstrated a SSRI exceeding 26%, indicating a substantial improvement in the acne. Massive proliferation of C. acnes is considered a prominent feature of microbiota imbalance in acne skin, and porphyrins secreted by C. acnes can stimulate the inflammation of hair follicles and sebaceous glands to aggravate acne [38, 39]. Interestingly, we also observed reduced porphyrin levels after treatment by VISIA analysis, suggesting that EVE‐CHARM anti‐acne lotion regulates skin microecology and promotes skin barrier repair. More importantly, this topical postbiotic formulation works through a biological mechanism to address the root causes of acne, safer and more effective than traditional therapeutic drugs. With suitable skin care, no significant adverse effects during treatment.

**TABLE 2:** Stability assessment.

Items	Test condition	Test sample	1‐month results
Light	The test materials were placed in a light box with fluorescent lamps and monitored once a week.	Five bottles each of the lyophilized powder and the accompanying solution	The product exhibits no discoloration, emits no odor, contains no impurities, and the packaging is securely sealed.
Cold	The test materials were placed in the refrigerator (−8°C to −10°C) and monitored once a week.	Five bottles each of the lyophilized powder and the accompanying solution	The product exhibits no discoloration, emits no odor, contains no impurities, and the packaging is securely sealed.
Heat	The test materials were placed in a thermostat (48°C to 50°C) and monitored once a week.	Five bottles each of the lyophilized powder and the accompanying solution	The product exhibits no discoloration, emits no odor, contains no impurities, and the packaging is securely sealed.
High‐low temperature	The test materials were placed in the high‐low temperature test chamber (−10°C to −48°C) and monitored once a week.	Five bottles each of the lyophilized powder and the accompanying solution	The product exhibits no discoloration, emits no odor, contains no impurities, and the packaging is securely sealed.
Ambient	The test materials were placed at normal room temperature and monitored once a week.	Five bottles each of the lyophilized powder and the accompanying solution	The product exhibits no discoloration, emits no odor, contains no impurities, and the packaging is securely sealed.

Therefore, we can preliminarily propose the feasibility of EVE‐CHARM antiacne lotion combined with microneedling treating mild‐to‐moderate facial acne vulgaris, which might be attributed to its ability to restore skin microbiota imbalance and the skin barrier to reduce sebum production and inhibit inflammation. This study provides a new combination therapy for acne treatment, but additional samples are needed for further validation.

## 5 Conclusion

This study investigated the efficacy of a postbiotic formulation EVE‐CHARM antiacne lotion combined with microneedling for mild‐to‐moderate facial acne vulgaris. The current results demonstrated this combination treatment can manage skin lesions, reduce sebaceous gland secretion, and alleviate inflammation, which might be ascribed to the beneficial effects of postbiotics on the balance of the skin microbiota and the integrity of the skin barrier. Therefore, EVE‐CHARM anti‐acne lotion represents a valuable nonpharmacological intervention that offers an innovative therapeutic alternative for the prevention and treatment of acne vulgaris.

## Author Contributions

Conceptualization: Z.L., R.Y., and Q.M. Methodology: X.M., H.W., and H.C. Validation: P.L., Y.X., and C.Y. Formal analysis: Z.L., P.L., and Y.X. Investigation: J.Z., T.L., X.L., and J.Z. Data curation: J.Z. and Z.L. Writing – original draft preparation: Z.L. Writing – review and editing: Z.L., Y.W., L.L., W.L., and Q.M. Visualization: Z.L. and C.Y. Supervision: J.Z., J.Z., and Q.M.

## Ethics Statement

Ethical approval was granted by the ethics committee of Guangzhou MLT Medical Cosmetic Clinic (No.2023002). All recruited subjects were aware of the study process and signed the written consent forms.

## Consent

For all personal information used in this article, for example, personal images, a consent for publication has been obtained.

## Conflicts of Interest

The authors declare no conflicts of interest.

## Data Availability

The data that support the findings of this study are available from the corresponding author upon reasonable request.
